# Anti-Insulin Immune Responses Are Detectable in Dogs with Spontaneous Diabetes

**DOI:** 10.1371/journal.pone.0152397

**Published:** 2016-03-31

**Authors:** Jong-Hyuk Kim, Eva Furrow, Michelle G. Ritt, Paul J. Utz, William H. Robinson, Liping Yu, Andrea Eckert, Kathleen Stuebner, Timothy D. O’Brien, Lawrence Steinman, Jaime F. Modiano

**Affiliations:** 1 Department of Veterinary Clinical Sciences, University of Minnesota College of Veterinary Medicine, St. Paul, MN, United States of America; 2 Animal Cancer Care and Research Program, University of Minnesota, St. Paul, MN, United States of America; 3 Masonic Cancer Center, University of Minnesota, Minneapolis, MN, United States of America; 4 Division of Immunology and Rheumatology, Department of Medicine, Stanford University School of Medicine, Stanford, CA, United States of America; 5 Institute for Immunity, Transplantation and Infection, Stanford University School of Medicine, Stanford, CA, United States of America; 6 Barbara Davis Center for Childhood Diabetes, University of Colorado School of Medicine, Aurora, CO, United States of America; 7 Clinical Investigation Center, University of Minnesota College of Veterinary Medicine, St. Paul, MN, United States of America; 8 Department of Veterinary Population Medicine, University of Minnesota College of Veterinary Medicine, St. Paul, MN, United States of America; 9 Stem Cell Institute, University of Minnesota, Minneapolis, MN, United States of America; 10 Department of Neurology and Neurological Sciences, Stanford University School of Medicine, Stanford, CA, United States of America; 11 Department of Pediatrics, Stanford University School of Medicine, Stanford, CA, United States of America; 12 Center for Immunology, University of Minnesota, Minneapolis, MN, United States of America; La Jolla Institute for Allergy and Immunology, UNITED STATES

## Abstract

Diabetes mellitus occurs spontaneously in dogs. Although canine diabetes shares many features with human type-1 diabetes, there are differences that have cast doubt on the immunologic origin of the canine disease. In this study, we examined whether peripheral immune responses directed against islet antigens were present in dogs with diabetes. Routine diagnostics were used to confirm diabetic status, and serum samples from dogs with (N = 15) and without (N = 15) diabetes were analyzed for the presence of antibodies against islet antigens (insulin, glutamic acid decarboxylase, insulinoma-associated protein tyrosine phosphatase, and islet beta-cell zinc cation efflux transporter) using standard radioassays. Interferon-γ production from peripheral blood T cells stimulated by porcine insulin and by human insulin was tested using Elispot assays. Anti-insulin antibodies were detectable in a subset of diabetic dogs receiving insulin therapy. Pre-activated T cells and incipient insulin-reactive T cells in response to porcine or human insulin were identified in non-diabetic dogs and in dogs with diabetes. The data show that humoral and cellular anti-insulin immune responses are detectable in dogs with diabetes. This in turn provides support for the potential to ethically use dogs with diabetes to study the therapeutic potential of antigen-specific tolerance.

## Introduction

Spontaneous diabetes mellitus is common in dogs, with a consistent worldwide incidence [1]. Like human type-1 diabetes (T1D), canine diabetes is characterized by erosion of pancreatic beta cells, pancreatic islet degeneration, and insulin-dependence [2–4]. The inability to achieve satisfactory clinical control of canine diabetes without administration of exogenous insulin indicates that this is an insulin-dependent disease.

The causes of diabetes in dogs remain incompletely understood and may be heterogeneous. There are strong breed predispositions [2, 5], indicating there are genetic or heritable components of this disease. These have been traced to polymorphisms in the dog leukocyte antigen (DLA) locus of the major histocompatibility complex (MHC) [6], as well as in genes that regulate immune and endocrine function [7], including the gene encoding insulin itself [8].

These genetic characteristics of canine diabetes strongly resemble those of human T1D [9–11]. But despite these similarities and relation to immune-specific genes, the roles of the immune system in the etiology of canine diabetes, and in the development of therapeutic resistance remain unresolved. Insulin autoantibodies (IAA) [12], glutamic decarboxylase antibodies (GADA), and insulinoma-associated protein -2 antibodies (IA-2) [13] have been reported in diabetic dogs, but recent studies suggest that canine diabetes is not exclusively an autoimmune condition [4, 14].

Here, we examined whether peripheral immune responses directed against islet antigens were present in dogs with diabetes. We demonstrate that humoral and cellular immune responses against insulin were detected in a subset of diabetic dogs treated with exogenous insulin, suggesting that approaches to induce antigen-specific tolerance could be an option to improve glycemic control of dogs with diabetes.

## Materials and Methods

### Dog recruitment and sample collection

Two cohorts of dogs were used for this study. One cohort consisted of 19 dogs (9 non-diabetic; 10 diabetic) recruited for an ongoing study of urinary stone formation. This study was approved by the University of Minnesota Institutional Animal Care and Use Committee (protocol 1207A17243). Owner consent included permission to use samples for additional studies, including the present assessment of diabetes status. Whole blood was collected by peripheral venipuncture and evacuated into sterile clot tubes to isolate serum. Sera were processed routinely and stored at -80C. The other cohort consisted of 11 dogs (6 non-diabetic; 5 diabetic) recruited through the internal medicine and general practice services, and from the employee companion dog population of the University of Minnesota Veterinary Medical Center. Samples were obtained under the supervision of the University of Minnesota Institutional Animal Care and Use Committee (protocol 1304-30546A) with informed consent from the owners, including an incentive for participation.

Two of the dogs in cohort-1 had a history of concurrent pancreatitis based on abdominal ultrasound imaging and SNAP canine pancreas-specific lipase (cPL) testing. Two non-diabetic dogs in cohort-1 also had pancreatitis without evidence of hyperglycemia or clinical signs of diabetes. One of the diabetic dogs in cohort-2 had congenital beta cell aplasia (juvenile onset diabetes). None of the dogs in either cohort had clinical or laboratory evidence of concurrent Cushing’s disease (hyperadrenocorticism). Thus, all but possibly three of the affected dogs in the study had idiopathic diabetes [2].

Whole blood was collected as above and evacuated into clot tubes to isolate serum and into CPT separator tubes (BD, Franklin Lakes, NJ) to isolate peripheral blood mononuclear cells (PBMCs). Sera were processed routinely and stored at -80C.

Enrollment in the study did not require modification of treatment for diabetes or other diseases. The dogs’ performance status was monitored based on maintenance of body weight, results of fructosamine (glycated serum proteins) tests, serial blood glucose concentrations, results of 12–24 hour glucose curves, and owners’ perception of clinical signs attributable to diabetes (appetite, thirst and urine production, activity levels) [15]. Blood glucose concentrations were measured using routine methods with the i-STAT^®^ System (Abbott Point of Care, Inc., Abbott Park, IL) or the Beckman Coulter AU Chemistry Analyzer (Beckman Coulter, Inc., Brea, CA). Diabetic control was rated as “good”, “fair”, or “poor” based on these parameters. Dogs with serum fructosamine concentrations between 250 and 350 μmol/L, 12–24 hour blood glucose curves showing a nadir between 80 and 150 mg/dL, average blood glucose <300 mg/dL, stable body weight or weight gain, and owner-reported observations of normal appetite, water consumption and urine production were classified as having good control. Dogs with improvement of clinical signs and stable body weight, but with incomplete control of their diabetes based on serum fructosamine concentrations >350 μmol/L, blood glucose curves showing a nadir >150 mg/dL, average blood glucose >300 mg/dL were classified as having fair control. Dogs with no clinical improvement with insulin therapy were classified as having poor control.

### Peripheral blood mononuclear cell preparation

PBMCs were isolated by discontinuous density centrifugation. Approximately 10 mL of whole blood were processed from each dog with yields of 5x10^5^–5x10^6^ PBMCs. PBMC isolation was done as soon as possible after collection, as the viability decreased by about 50% for every two hours that samples remained in the CPT tubes. PBMCs were washed and cryopreserved in liquid nitrogen in a solution consisting of 90% fetal bovine serum (FBS, Atlas Biologicals, Fort Collins, CO) and 10% dimethyl sulfoxide until further analysis.

### Quantification of anti-islet antibodies

Serum samples from 31 dogs were analyzed for the presence of anti-islet antibodies. This analysis included measurement of anti-insulin antibody using the current standard micro IAA radioassay (mIAA), anti-IA-2 antibody, anti-GAD, and anti-ZnT8 antibody as described [16, 17].

### Quantification of anti-insulin T cell responses

PBMC samples were collected from pet dogs over the span of six weeks. Samples were stored in liquid nitrogen for 36–42 weeks prior to conducting the assays. PBMC were recovered from liquid nitrogen by rapid thawing to 37°C in a water bath followed by washing twice in pre-warmed RPMI-1640 medium supplemented with 10% FBS. The yield and viability from all the cryopreserved PBMC samples was approximately 90%. PBMC were cultured in complete RPMI-1640 medium supplemented with 10% FBS, 2 mM L-glutamine, 2 mM sodium pyruvate, 0.1% Primocin (Invivogen, San Diego, CA), and 10 mM 4-(2-hydroxyethyl)-1-piperazine ethanesulfonic acid buffer (HEPES) and rested overnight before stimulation.

An Elispot kit (R&D Systems, Minneapolis, MN) was used to quantify T cell activation. We first verified whether stimulation with Concanavalin A (ConA, 2.5–5 μg/mL, Sigma-Aldrich, St. Louis, MO) for 24 hr was suitable to induce measurable levels of interleukin-2 (IL-2) in fresh PBMC from unaffected dogs. Next we examined the capacity of PBMC to respond to ConA (2.5 μg/mL) upon recovery from cryopreservation. Fresh PBMC from two dogs used in the validation process described above and PBMC recovered from cryopreservation from two unaffected control dogs (MD 09 and MD 10) were stimulated as described above to examine interferon-γ (IFN-γ) production.

We used the IFN-γ assay to determine if T-cells recognizing insulin were present in PBMC from five unaffected dogs and four dogs with diabetes. PBMC (2 x 105/well) were stimulated for 24 hr using ConA and a commercial canine polyvalent vaccine containing modified live canine distemper virus, canine adenovirus type-2, and canine parvovirus (Nobivac Canine 3-DAPV, Merck Animal Health, Millsboro, DE) as positive controls for detection of recall antigens [18]. Human insulin and porcine insulin as the test antigens were incubated for 24 hr. Both insulins were used at 5 μg/mL to provide antigen saturation. Reagents used for stimulation were diluted in sterile phosphate buffered saline (PBS), and an equivalent amount of PBS as negative control was added to the unstimulated controls to establish a background, or the presence of pre-activated cells secreting IFN-γ. Assays were done in duplicate and spots were quantified manually under a dissecting microscope according to the manufacturer’s protocol. Any visible spot that was round to oval and had a dark center was scored, regardless of whether the borders were sharp or “fuzzy”. Spots were not further characterized based on size or morphology, but streaks, lines, or amorphous spots were not counted.

### Statistics

Descriptive statistics (mean, median, standard error, standard deviation) were generated using GraphPad Prism 6 (GraphPad Software, Inc., San Diego, CA). The Mann Whitney test was used for comparison of skewed data (blood glucose concentrations). The Fisher’s exact test was used to determine if variables had different frequencies between groups (presence of anti-insulin antibody in non-diabetic and diabetic groups). The Pearson correlation coefficient (Pearson’s r) was used to determine the strength of linear correlation between variables. A p-value < 0.05 was considered significant.

## Results

### Demographics of the population

Table 1 shows the demographic characteristics of the diabetic and non-diabetic dogs from which we obtained samples for this study. Ten dogs from the first cohort had a confirmed diagnosis of diabetes, and the other nine were selected among those that had spot glucose concentrations within the reference range to match the breed and age of the diabetic dogs. Samples from one dog in the diabetic population were collected prior to and after the onset of diabetes. When the pre-diabetic sample was collected, the dog had mild hyperglycemia (142 mg/dL) but no clinical signs of diabetes; when the subsequent sample was collected one month later, the dog’s clinical condition had progressed to overt diabetes (serum glucose = 374 mg/dL); thus, it was included with the diabetic dogs for analysis. One of the non-diabetic dogs also was hypothyroid. Five dogs from the second cohort had a diagnosis of diabetes that was verified through medical records and by assessment of spot glucose levels (N = 4) or based on its medical record (N = 1). The six non-diabetic dogs in this second cohort were collected during the same time frame as the diabetic dogs. Fourteen of the 15 diabetic dogs in total (both cohorts) were on maintenance insulin therapy. Of these, eleven dogs were receiving human neutral protamine Hagedorn (NPH) insulin. The dose and schedule for 10 of these dogs was between 0.8 and 1.1 international units (IU)/kg given subcutaneously every twelve hours. One dog was receiving 0.25 IU/kg subcutaneously every twelve hours. Two dogs were receiving protamine zinc insulin (PZI, 0.6 IU/kg and 1 IU/kg) subcutaneously every twelve hours, and one dog was receiving Vetsulin (porcine insulin for veterinary use, 0.8 IU/kg) subcutaneously every twelve hours. Diabetic control was considered “good” in seven dogs, “fair” in four dogs, and “poor” in four dogs. All of the dogs that had non-diabetic conditions were receiving appropriate treatment (Table 1).

**Table 1:** Demographic Characteristics of Dogs in Study.

	Non-diabetics	Diabetics
All dogs	N = 15	N = 15
**Breeds**	Australian shepherd, Bichon Frise, Great Dane, Greyhound, Labrador retriever, Newfoundland, Siberian husky, Miniature schnauzer (5), Mixed breed (1), Shih-tzu	Avon terrier, American Eskimo, Beagle, Bichon Frise, Lhasa Apso, Miniature schnauzer (5)*, Mixed breed (2), Pug, Samoyed, Shih-tzu
**Mean Age (Years ±SD)**	9.2 (1.7)	9.9 (2.9)
**Median Age (Years)**	9	10
**Intact Males**	1	0
**Neutered Males**	12	10
**Intact Females**	0	0
**Spayed Females**	3	5
**Mean Blood glucose (mg/dL ±SD)** †	106.8 (14.3)	343.5 (180.7)
**Median Blood glucose (mg/dL)** †	105	347.5
**Co-morbidities**	Hypothyroidism, Pre-diabetic hyperglycemia	Hypothyroidism
**Treatment**	Pancreatic enzymes, Physostigmine, Levothyroxine	Levothyroxine, NPH Insulin (11), PZI Insulin (2), Vetsulin
**mIAA positivity** ‡	0/16	5/15

*Samples from one miniature schnauzer were collected prior to confirmation of diabetes and after confirmation of diabetes. This dog was only included in the diabetic group for analysis

^†^Significantly different between groups (p = 0.0001)

^‡^Significantly different between groups (p = 0.042)

**Fig 1:**
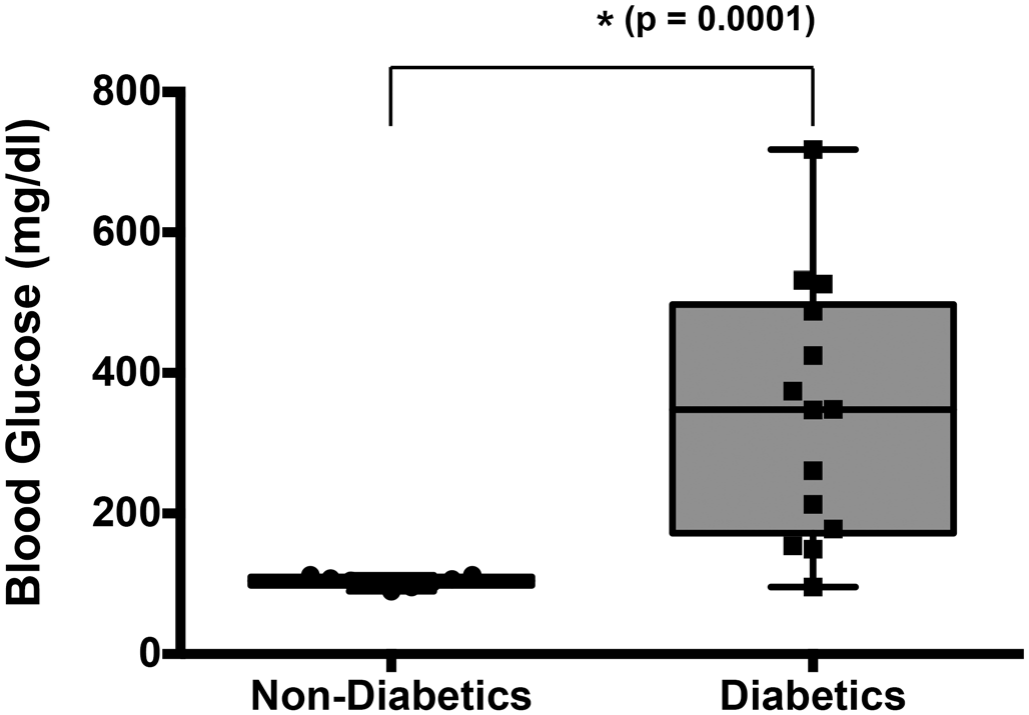
Blood glucose levels in diabetic and non-diabetic dogs. Blood glucose was measured as described in Materials and Methods. Box plot shows blood glucose concentrations for non-diabetic and for diabetic dogs (median and the 25th to 75th percentiles; whiskers represent outliers), as well as individual data points. The blood glucose concentrations in dogs with diabetes were significantly higher that those in dogs without diabetes (p = 0.0001, Mann Whitney test).

Despite the insulin treatment and independent of their clinical diabetic control status, all but one dog in the diabetes group were hyperglycemic at the time of sample collection, and blood glucose concentrations were significantly different (p = 0.0001, Mann Whitney test) between the diabetic and the non-diabetic groups (Fig 1).

### Anti-insulin antibodies are detectable in a subset of dogs with diabetes

**Fig 2:**
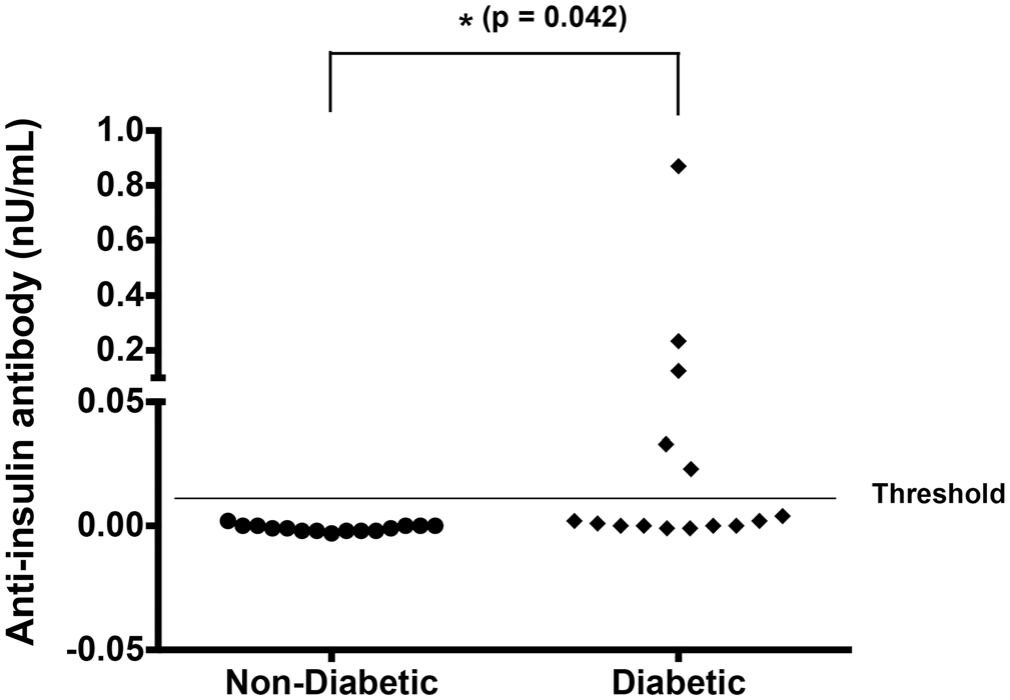
Anti-insulin antibodies in diabetic and non-diabetic dogs. Anti-insulin antibodies were measured using the microIAA radioassay. Scatter plot showing individual anti-insulin antibody concentrations (in nU/mL) for non-diabetic dogs and for diabetic dogs. The gray line at 0.01 nU/mL represents the upper threshold for negative results. The frequency of diabetic dogs with anti-insulin antibody above threshold was significantly different from that of the non-diabetic dogs (p = 0.042, Fisher’s exact test).

The concentration of IAA was above the positive threshold in 5 of 15 diabetic dogs, and it was not detectable in any of the 15 non-diabetic dogs (Table 1 and Fig 2) (p = 0.042, Fisher’s exact test). Antibodies against IA-2 and ZnT8 were not detected in any of dogs, and consistent with results from a recent study of 121 dogs with diabetes [14], we also did not find anti-GAD antibodies in any of diabetic dogs in these cohorts, although three of 15 non-diabetic dogs had detectable anti-GAD. No anti-islet antibodies were detectable in serum from the dog whose samples were collected when it was pre-diabetic and after it developed overt diabetes.

### Pre-activated T cells and incipient, insulin-reactive T cells are present in peripheral blood of non-diabetic dogs and dogs with diabetes

The amino acid sequences of human, canine, and porcine pre-proinsulin show moderate divergence in the signal peptide and the C-peptide domains [19]. However, the sequence for the insulin-A chain is identical in the three species, and the sequence for the insulin B chain differs only by a single amino acid in human (T30) vs. dog and pig (A30) [20]. To determine if insulin induces peripheral antigen-specific immune response, we tested activation of T cells obtained from PBMC treated with insulin.

**Fig 3:**
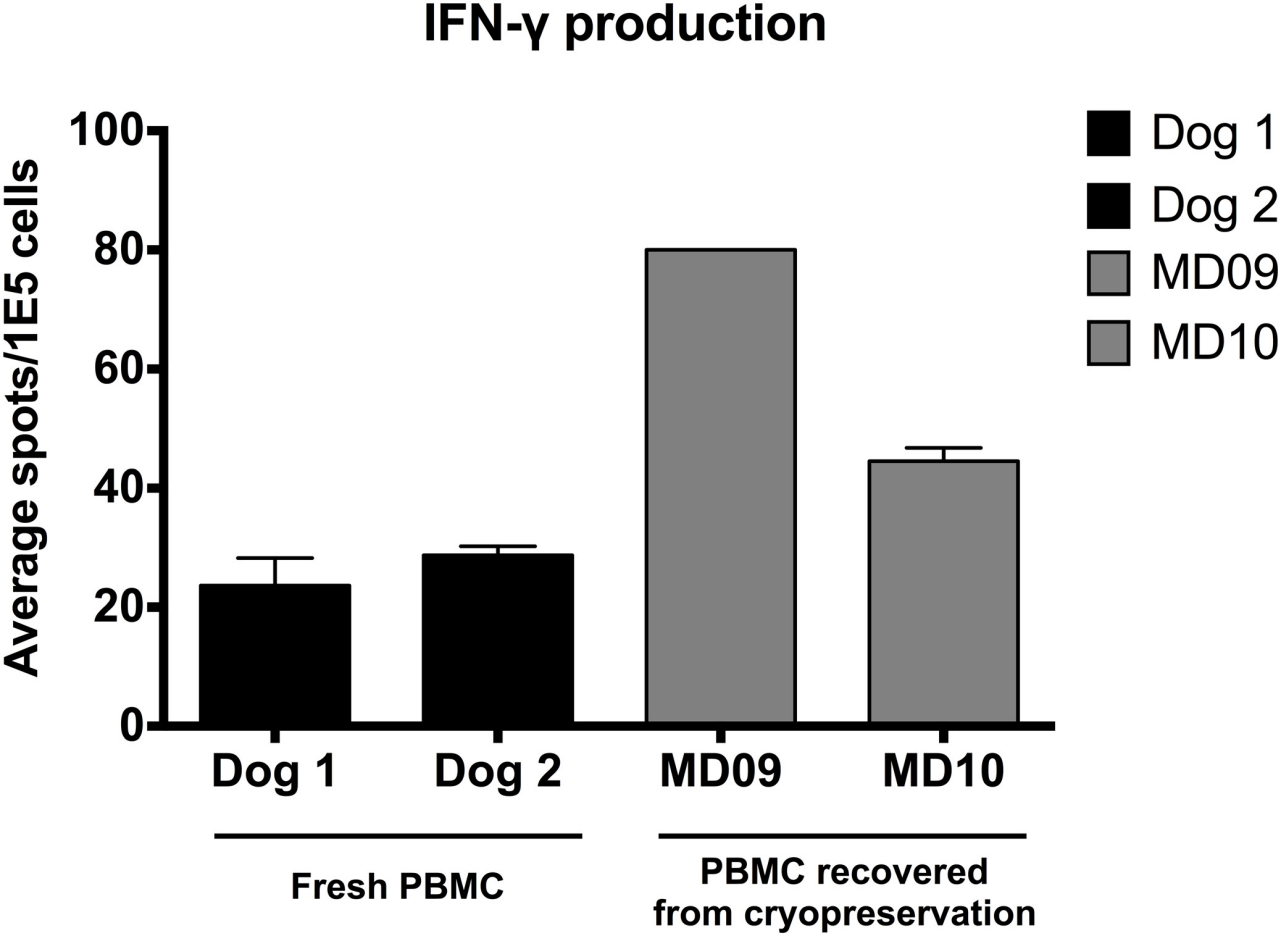
Interferon-γ (IFN-γ) production of fresh and cryopreserved canine PBMC in response to ConA. Fresh PBMC from two unaffected dogs (Dog 1 and Dog 2) and PBMC recovered from cryopreservation from two unaffected dogs (MD 09 and MD 10) were stimulated with Con A (2.5 μg/mL). Bar graphs shows IFN-γ production (mean ± SD of the number of spots per 10^5^ cells). No spots were seen from any of the unstimulated PBMC samples in this assay.

We first validated that ConA stimulation in fresh PBMC from unaffected dogs induced measurable levels of interleukin-2 (IL-2). The response to ConA was maximal at 2.5 μg/mL using 1–2 x 10^6^ PBMC/mL (1–2 x 10^5^ PBMC/well), with responses in the range of 50–100 spots/well. Fresh canine PBMC (Dog 1 and Dog 2) and cryopreserved PBMC (MD 09 and MD 10) from unaffected dogs also retained the capacity to elaborate IFN-γ in response to ConA in the range of 20–80 spots/10^5^ cells (Fig 3). No spots were seen from any of the unstimulated PBMC samples or from the background samples in this assay. PBMCs from nine of 10 dogs showed an increase (range = 7 to 1,708-fold) in the frequency of IFN-γ-producing cells after ConA stimulation. In addition, T-cells from five of eight dogs also elaborated IFN-γ in response to stimulation by the DAPV vaccine, indicating that IFN-γ production could be used as a surrogate to identify T-cells with reactivity against recall antigens.

**Fig 4:**
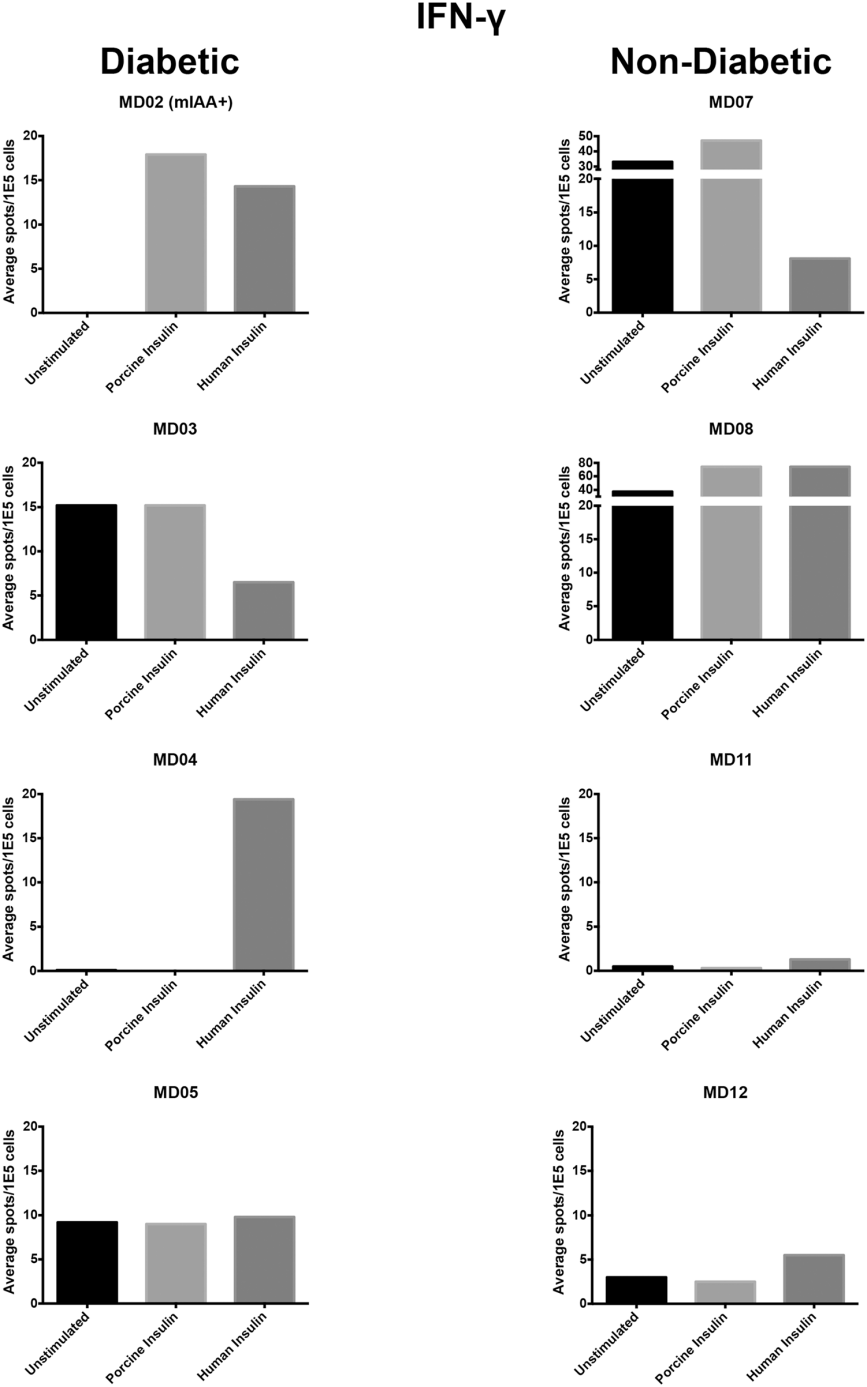
Interferon-γ production by insulin-stimulated canine PBMC. PBMC were collected, cryopreserved, recovered, and stimulated as described in Materials and Methods. Bar graphs shows IFN-γ production by unstimulated and insulin-stimulated PBMCs for the four non-diabetic dogs and the four diabetic dogs in which cytokine production was measured.

We used this Elispot assay to identify insulin-specific T cells in peripheral blood from diabetic (N = 4) and non-diabetic dogs (N = 4). IFN-γ responses were detectable in two diabetic dogs after insulin stimulation (Fig 4). The dog with the most robust anti-insulin T-cell response (MD 02) also had detectable anti-insulin antibodies (0.234 nU/mL), and its diabetes was poorly controlled on NPH insulin. This dog had no pre-activated T cells; its PBMCs showed appropriate polyclonal (854 spots per 100,000 cells, respectively) and recall antigen (77 spots per 100,000 cells, respectively) stimulation; and its T-cells reacted against both porcine insulin (18 spots per 100,000 cells) and human insulin (14 spots per 100,000 cells). The other diabetic dog that showed anti-insulin T-cell responses (MD 04) did not have detectable anti-insulin antibodies in its serum; its PBMCs responded primarily against human insulin, and curiously, they showed a blunted polyclonal response to ConA.

Two non-diabetic dogs showed a response against insulin (MD 07 and MD 08), but both had elevated background responses (>30 spots per 100,000 cells without exogenous stimulation), suggesting T cells were pre-activated in vivo or during the processing steps. The response against insulin in these dogs increased by only two-fold over background, as compared to the infinite (divided by 0) and the 194-fold increase over background seen in the diabetic dogs in which no pre-activated T cells were detectable.

## Discussion

The role of autoimmunity in the etiology of canine diabetes remains incompletely understood. Here, we show that peripheral humoral and cellular immune responses against insulin are detectable in dogs with diabetes that were treated with exogenous insulin.

Circulating autoantibodies are a hallmark of autoimmune disease, including human T1D [9–11, 21]; however, there is a debate regarding the frequency of autoantibodies and their usefulness as a maker for canine diabetes [14]. Our results show that, as in humans [22], anti-insulin responses in dogs are heterogeneous. Anti-insulin antibodies were only present in five of fifteen insulin-treated diabetic dogs (33%), and we cannot distinguish if the antibodies represent a response against an endogenous insulin autoantigen or a response against exogenous (therapeutic) insulin. Previously, Davison et al. reported that autoantibodies against insulin were detectable in five out of 40 newly diagnosed, untreated diabetic dogs in one study, and autoantibodies against proinsulin were detectable in eight out of 15 newly diagnosed diabetic dogs in another [23, 24], and while these responses also were heterogeneous, they indicated that either proinsulin, insulin, or both can act as autoantigens.

Autoreactive T cells play important roles in the pathogenesis of T1D in humans and in NOD mice [25–28]. A potential role for autoreactive T cells in the pathogenesis of canine diabetes also has been suggested based on the association of the disease with MHC class II gene haplotypes [6] and with certain polymorphisms of T cell cytokine genes and the *CTLA4* promoter [29, 30]. Our data show that insulin-specific T cells are detectable in a subset of diabetic dogs, although incipient, insulin-reactive T cells also might be present in non-diabetic dogs.

Finally, our results also support the notion that canine diabetes is a heterogeneous disease. A recent study showed that human T1D could be separated into distinct immunological phenotypes based on blood autoimmune response and pancreatic islet histopathology [22]. Thus, measuring T cell activation parameters might be useful to establish comparable phenotypes in dogs with diabetes mellitus and to refine the classification of this disease and its potential similarities to the corresponding immunologic phenotypes of human T1D.

## Conclusions

We show here that anti-insulin antibodies and insulin-reactive T cells are detectable in a subset of diabetic dogs on insulin therapy. The data suggest that anti-insulin immune responses might contribute to the pathogenesis and/or the progression of treated canine diabetes, creating an opportunity to ethically examine the therapeutic potential of antigen specific tolerization in a clinically realistic model.

## Supporting Information

S1 File Arrive Guidelines Checklist. This document provides documentation for compliance with the Animal Research: Reporting In Vivo Experiments, including the location of relevant information in the submitted manuscript.
(PDF)
